# Effect of Commercially Available Egg Cures on the Survival of Juvenile Salmonids

**DOI:** 10.1371/journal.pone.0021406

**Published:** 2011-06-27

**Authors:** Shaun Clements, Rob Chitwood, Carl B. Schreck

**Affiliations:** 1 Oregon Department of Fish and Wildlife, Corvallis, Oregon, United States of America; 2 Department of Fisheries and Wildlife, Oregon State University, Corvallis, Oregon, United States of America; 3 Oregon Cooperative Fish and Wildlife Research Unit, United States Geological Survey, Oregon State University, Corvallis, Oregon, United States of America; Institute of Marine Research, Norway

## Abstract

There is some concern that incidental consumption of eggs cured with commercially available cures for the purpose of sport fishing causes mortality in juvenile salmon. We evaluated this by feeding juvenile spring Chinook (*Oncorhynchus tshawytscha*) and steelhead (*O. mykiss*) with eggs cured with one of five commercially available cures. We observed significant levels of mortality in both pre-smolts and smolts. Depending on the experiment, 2, 3, or 4 of the cures were associated with mortality. Mortality tended to be higher in the smolts than in the parr, but there was no clear species effect. The majority of mortality occurred within the first 10 d of feeding. Removal of sodium sulfite from the cure significantly reduced the level of mortality. Soaking the eggs prior to feeding did not reduce mortality. We observed a clear relationship between the amount of cured egg consumed each day and the survival time. We conclude that consumption of eggs cured with sodium sulfite has the potential to cause mortality in juvenile steelhead and Chinook salmon in the wild.

## Introduction

Anglers in the Pacific Northwest and the Midwest states of the U.S. often use eggs that are cured with a mix of preservatives, dyes, salts, and sugars as bait for trout and salmon. These eggs may either be purchased pre-cured or anglers can purchase the cures or cure ingredients and prepare their own eggs. Although anglers are typically targeting adult fish, juvenile salmonids are known to consume cured eggs incidentally (Scott Amerman: guide and cured egg manufacturer, Pers. Comm.). In the Pacific Northwest states, anglers typically cure the full ovary and cut it into smaller pieces which are attached to a hook. Conversely, anglers in the Midwest of the US (Great Lakes) often use net bags (spawn sacks) to contain the eggs, though there is likely still some level of exposure to smolts from chumming and/or splitting of the bags (Jay Wesley, Lake Michigan Basin Coordinator, Michigan Department of Natural Resources, Pers. Comm.). The level of exposure experienced by individual juvenile fish is unknown, but in areas of heavy fishing pressure it is potentially quite high. Factors such as the number of anglers fishing cured eggs, the time spent fishing in the home range or vicinity of an individual fish, the appetite of the fish, and the density of fish in an area could all affect the level of exposure.

Despite the potential for exposure, there has been little consideration for the effects of cured eggs on the health of juvenile salmonids. It has generally been assumed that the consumption of cured eggs has little effect on juveniles. However, at least some of the ingredients used in these cures are known to have adverse effects on vertebrates. For example, the majority of commercially available cures contain sulfite compounds, typically sodium sulfite. Sulfites and sulfite radicals, the intermediate products of sulfite metabolism, cause damage to nucleic acids, proteins, and lipids in mammals [1]–[4] and have been shown to cause mortality in teleosts [5], [6]. Another commonly used group of compounds, nitrites, also have a number of negative effects, including decreased growth in teleosts [7]. Water borne exposure to sodium nitrite, the most commonly used form of nitrite, has also been shown to cause mortality in salmonids [8], [9].

Given the concerns about the viability of many wild populations of salmonids, it is important to minimize the risks encountered by these fish wherever possible. Our objective was to determine whether ingestion of eggs cured with commercially available cures affected the survival of juvenile salmonids. We used a variety of approaches, including feeding trials and oral administration trials, to determine whether these cures had the potential to cause mortality in juvenile salmonids. In addition, we assessed whether mortality associated with consumption of cured eggs was caused by sodium sulfite.

## Methods

Ethics Statement: Animals were treated in accordance with the principles and procedures of the Laboratory of Animal Resources Center (LARC) at OSU. All manipulations in this manuscript were approved by the LARC prior to experimentation (permit number 3818).

### 2.1 Fish

We used juvenile spring Chinook salmon, *Oncorhynchus tshawytscha* (North Santiam River stock), and steelhead trout, *Oncorhynchus mykiss* (Alsea River stock). All fish were held under ambient photoperiod in 1500 L circular tanks (the two species were held separately) at Oregon State University's (OSU) Fish Performance and Genetics Laboratory. Pathogen free, flow through water (∼12°C) was supplied from a well. The stock fish were fed twice daily with semi-moist pellet (Bio-Oregon, Skretting Inc., Vancouver, Canada). All fish were apparently healthy throughout the trials and we had no incidence of disease. All experiments were conducted between November 2008 and June 2010. A summary of the experiments is given in Table 1.

**Table 1:** Description of experimental methods.

experimental description	species	life-stage	cures tested	feeding method	# treatment groups	number of tanks	fish/tank	duration of test (d)	description of treatment
2.3	CHS	Parr	1–4	by hand	5	15	55	23	Fed cures 1–4 at 1.5% BW d
2.3	CHS	Smolt	1–5	by hand	5	18	55	23	Fed cures 1–5 at 1.5% BW d
2.3	ST	Parr	1–4	by hand	6	15	55	23	Fed cures 1–4 at 1.5% BW d
2.3	ST	Smolt	1–5	by hand	6	18	55	23	Fed cures 1–5 at 1.5% BW d
2.4	CHS	Parr	1	by hand	3	6	30	10	Fed full cure, cure without SS, or cure without SS and sodium nitrite
2.4	CHS	Parr	5	by hand	2	4	30	10	Fed full cure or cure without SS
2.4	CHS	Parr	1	syringe	2	1	20	10	Administered full cure or cure without SS
2.4	CHS	Parr	5	syringe	2	1	20	10	Administered full cure or cure without SS
2.5	CHS	Parr	4	syringe	8	2	40	10	Administered the equivalent of 3, 6, or 10 eggs, with or without pellets
2.5	CHS	Parr	5	syringe	8	2	40	10	Administered the equivalent of 3, 6, or 10 eggs, with or without pellets
2.6	CHS	Parr	1	syringe*	2	2	20	10	Uncured or cured lipid mix applied to gills
2.7	CHS	Parr	1	by hand	4	12	30	10	Fed cured eggs soaked for 0, 30, or 60 s or 10 min

Experimental description refers to the section number in the methods section. CHS: Spring Chinook, ST: Steelhead.

### 2.2 Cured egg preparation

We stripped fresh eggs from spring Chinook collected at the Oregon Department of Fish and Wildlife's (ODFW) Clackamas Fish Hatchery and steelhead collected at the Alsea Fish Hatchery. The spring Chinook eggs were contained within a skein whereas the steelhead eggs were ovulated, and so were loose. The eggs were frozen in vacuum sealed bags within 2 h of collection and stored at −4°C until use. We purchased four commercially available cures from local stores and prepared the eggs as follows. We thawed sufficient eggs for 5 d and stripped the spring Chinook eggs from the skeins prior to curing. The eggs were then divided among a negative control and four treatment groups. The eggs for the four treatment groups were cured using the commercially available cures (cures 1–4), following the manufacturers' instructions. Typically, this involved adding the cure at a ratio of between 1∶9 and 1∶32, mixing the eggs thoroughly, and leaving them to cure for 12–24 h before use. In addition, we tested another brand of cure, sold in premixed form (cure 5), during some of the experiments. The cured and uncured eggs were stored at 4°C prior to use. During each experiment, we prepared a fresh batch of cured eggs every 4–5 d to ensure that they remained fresh.

### 2.3 Effect of consuming cured eggs on the survival of juvenile salmon

We evaluated the effects of the cured eggs on two lifestages (parr and smolt) and in two species (*O. mykiss* and *O. tshawytscha*). We did not specifically test for smoltification during this experiment. However, the experiments were timed to coincide with the period of release and migration to the ocean of these stocks.

We collected juvenile steelhead or Chinook from the stock tank using a dipnet. The fish were then randomly assigned to one of 18 (pre-smolt) or 21 (smolt) tanks (336 L; N = 55/tank). The experimental tanks were supplied with flow through well water (∼12°C). We measured the total biomass in each tank prior to stocking. The fish were acclimated to the experimental tanks for 4 d and fed the same pellet diet as in the stock tanks. Following this, the tanks were randomly divided into 6 (presmolt) or 7 (smolt) groups (3 tanks per group) consisting of a control (fed standard pellet feed), a negative control (fed uncured salmon eggs), or treatment groups that were fed salmon eggs cured with 1 of 4 or 5 commercially available cures. The fish were fed daily (∼09:00) at 1.5% bodyweight (BW)/d for 23 d. We scored the feeding behavior of the fish in each tank on a scale of 1–4 based on the level of interest and the time taken to consume all the food: 1 = all food consumed within 10 s, 2 = all food consumed within 10–20 s, 3 = some food consumed within 2 min, 4 = no food consumed within 2 min. The feeders were not told which cures were being fed. However, there were obvious differences in the color and/or texture of the different cures. We monitored mortality and morbidity daily and adjusted the feed amount accordingly. We performed a post-mortem analysis of each fish that died, recording length, weight, sex, amount of food in the gut (broken and unbroken eggs), and general notes on tissue breakdown, lesions etc. We also measured the weight and length of survivors at the end of the experiment.

### 2.4 Effect of sodium sulfite on the survival of juvenile spring Chinook

At the completion of the experiments described above, we entered into an agreement with the cure manufacturers to obtain their cure formula. In exchange, ODFW agreed not to disclose the names or formulas of the companies. Therefore, we refer to the cures as cure 1–5 throughout this manuscript. We subsequently obtained a list of ingredients from three cure manufacturers (cures 3–5). These three cures contained various amounts of: salt, sugar, sodium sulfite, calcium propionate, sodium nitrite, potassium sorbate, dyes, and jello. The amounts and combinations of chemicals used in each of the cures varied, though the majority of the cure consisted of salt, sugar, and sodium sulfite. The remaining ingredients each contributed less than 2% by weight to the cure. We were only provided anecdotal information regarding the ingredients in cure 1, though we do know that the cure contains sodium sulfite and sodium nitrite. Based on toxicity data for each of the known ingredients and the concentration at which each ingredient is used, we hypothesized that the most likely cause of mortality was exposure to sodium sulfite. To test this hypothesis, we asked the manufacturer of cure 1 to provide 2 additional cures, one that did not have sodium sulfite and one that did not have sodium sulfite or sodium nitrite. In addition, we prepared two cures (with and without Na_2_SO_3_) using ingredients that were provided by the manufacturer of cure 5. We chose these two cures as they had caused the highest mortality in the previous tests. We used two experimental approaches to determine the effect. In one set of experiments, the animals were fed as described above. In a second set of experiments the cure was administered directly into the gut via an oral injection. The second approach was used to ensure a known amount of the cure was consumed by all individuals.

We evaluated the two forms (with and without sodium sulfite) of cure 1 and cure 5 separately. In each test, juvenile Chinook were collected from the stock tank using a dipnet, randomly assigned to 1 of 6 tanks (336 L; N = 30 fish/tank), and acclimated as described above. The tanks were randomly divided into 3 (cure 1) or 2 (cure 5) treatment groups as follows. Cure 1: a group fed salmon eggs cured with the complete cure, a group fed the same cure without sodium sulfite, and a group fed the same cure without sodium sulfite and sodium nitrite (2 tanks/group). Cure 5: one group fed salmon eggs cured with the complete cure and a second group fed the same cure without sodium sulfite (3 tanks/group). In both trials the fish were fed at 1.5% BW/d for a period of 10 d. We monitored mortality and morbidity as described above. This experiment was conducted in September and October, 2009.

In addition to the feeding studies, we also directly administered cures 1 and 5 (both with and without sodium sulfite) using a syringe to ensure a known amount of cure was consumed. Juvenile Chinook were divided into 2 groups for each cure type (cure 1 and 5): 1) complete cure or 2) cure without Na_2_SO_3_. The fish were marked using a unique fin clip for each group, stocked into a single experimental tank (336 L, N = 20 fish/tank), and acclimated as above. We did not replicate this experiment because of LARC restrictions on the use of live animals. We prepared the cure by crushing the salmon eggs and separating the contents from the chorion using a strainer. The lipid was then divided into two subsamples. One of the subsamples was cured by adding the full cure at the appropriate ratio. A second subsample was cured using the modified cure (without sodium sulfite). We used approximately 1.5 times the recommended amount of cure to obtain a more concentrated solution that could be administered in a lower volume. The lipid/cure mixtures were then thoroughly mixed, divided into aliquots, and frozen at −4°C. On each day, the fish were anesthetized in a weak solution of MS-222 (25 mg/L buffered with 62.5 mg/L NaHCO_3_) and fed 0.5 ml (approximately equivalent to 1.5 eggs/d) of the frozen lipid/cure mixture using a tuberculin syringe that was inserted through the esophagus into the stomach. We checked for correct placement of the syringe in 5 individuals prior to beginning the experiment. We monitored mortality and morbidity as described previously. This experiment was conducted in October 2009.

### 2.5 The relationship between the number of cured eggs consumed, the consumption of alternative food, and the survival time in juvenile spring Chinook

We evaluated the effect of consuming the equivalent of 3, 6, or 10 eggs with or without pellets each day for 10 d. We collected juvenile spring Chinook from the stock tank and randomly assigned them to one of eight treatment groups (N = 10/treatment group) as follows: 1) control (C: uncured eggs only), 2) low dose (LD: equivalent to 3 eggs/d), 3) medium dose (MD: equivalent to 6 eggs/d), and 4) high dose (HD: equivalent to 10 eggs/d). Treatment groups 5–8 consisted of the same categories (control and low, medium, and high dose). However, these fish also received 0.28 g pellet/d (Bio-Oregon, Longview, WA, USA). The volume of egg lipid was reduced for these treatment groups to ensure the total energy content was similar between treatment groups 2–4 and 5–8. The fish in each treatment group were divided randomly into two tanks (N = 5 fish/treatment group/tank; N = 40 fish/tank). The fish in each treatment group were identified by a unique fin mark.

The lipid cure mixture was prepared as described above using the appropriate concentration of cure for the low, medium, and high dose groups based on the manufacturers' recommended rate of application and the average weight of an uncured steelhead egg (0.5 g). We prepared three stock solutions (low, medium, and high) for each cure. Each stock solution was divided into 100 aliquots (10 fish/10 d) which were loaded into syringes and stored at −4°C until they were used.

Following acclimation (described above) we administered the contents of a single syringe to each fish daily for 10 d as described in the previous experiment.

This experiment was conducted twice. We tested the effect of cure 5 during the first experiment and cure 4 during the second experiment. These represent cures associated with high (cure 5) and low (cure 4) levels of mortality in the previous experiments. This experiment was conducted in May 2010.

### 2.6 Effect of intra-buccal administration of the cure

To determine whether the mortality observed in previous experiments was caused by inadvertent exposure of the cure to the gills in a confined space (the experimental tanks), we administered the cure directly onto the gills. The lipid mixtures were prepared as follows. We obtained egg lipid by crushing the eggs. Half of this lipid extract was cured with 1.5 times the recommended amount of cure 1 and divided into 10 aliquots (∼60 ml/aliquot). The remaining uncured lipid was also divided into 10 aliquots. All 20 aliquots were frozen until use. Forty spring Chinook parr were divided amongst two tanks (336 L) and allowed to acclimate for 4 d. Following acclimation we began the treatments once daily for 10 d. Each day we thawed two of the aliquots (one cured and one uncured) and pre-loaded 40 1-ml tuberculin syringes with 0.5 ml of the lipid mixture (20 cured and 20 uncured). This volume is equivalent to approximately 1 egg (by weight) or 1.5 eggs (by concentration). The fish were then anaesthetized in a weak solution of MS-222 (25 mg L^−1^ MS-222 buffered with 62.5 mg L^−1^ NaHCO3) and the cure was syringed into the rear of the buccal cavity, anterior of the esophagus. We monitored mortality and morbidity daily. This experiment was conducted in November 2009.

### 2.7 Effect of soaking on the toxicity of the cured eggs

To determine whether the mortality observed in the feeding trials was an artifact of the feeding protocol, we soaked the eggs prior to feeding. This was intended to simulate the soaking that might occur in a typical fishing scenario. We collected juvenile spring Chinook from the stock tank and randomly assigned them to 1 of 12 experimental tanks (N = 30/tank). The tanks were then divided into four groups consisting of: 1) a control group that was fed unsoaked eggs and groups that were fed eggs soaked for 2) 30 s, 3) 1 min, or 4) 10 min prior to feeding. We placed the eggs in a black cotton mesh pouch prior to feeding. For those groups that received pre-soaked eggs the pouches were dipped into the tank and gently agitated to encourage “milking” of the eggs (washing the cure off the surface). After 1 min soaking, we generally did not observe any of the cure washing off the eggs, suggesting that the majority of surface cure had been removed. At the end of the allotted time the pouch was opened and the eggs fed as normal. All feeding was conducted between 0900 and 1000 hours. We collected uneaten eggs in the afternoon to determine whether there were any differences in the amount of food eaten after soaking. This experiment was conducted in November 2009.

### 2.8 Analysis

Proportion data were arcsine square root transformed. We tested normality of the data using a Kolmogorov-Smirnov test. Data that failed the test of normality were compared using Kruskal-Wallis One Way Analysis of Variance on Ranks. Data that passed the test of normality were compared using ANOVA. Differences among the treatment groups were analyzed using Tukey's post test (comparison of treatment group with the control). We evaluated the effect of removing sodium sulfite on mortality during feeding trails using ANOVA or students t-test (cure 1 and cure 5, respectively). We tested for the effect of tank and presence or absence of pellet feed on survival time using a Cox Stratified model. Fish that survived to the end of the experiment were assigned a censored survival time of 10 d. We then tested for the effect of dose (number of eggs consumed) on the survival time using a Gehan-Breslow Kaplan Meier Survival analysis. Differences among the different groups (control, low, medium, and high) were compared post hoc with a pairwise multiple comparison Holm-Sidak test. Differences that had a P value of <0.05 were considered significant.

## Results

### 3.1 Effect of cured egg mixtures on survival of juvenile salmon

**Figure 1:**
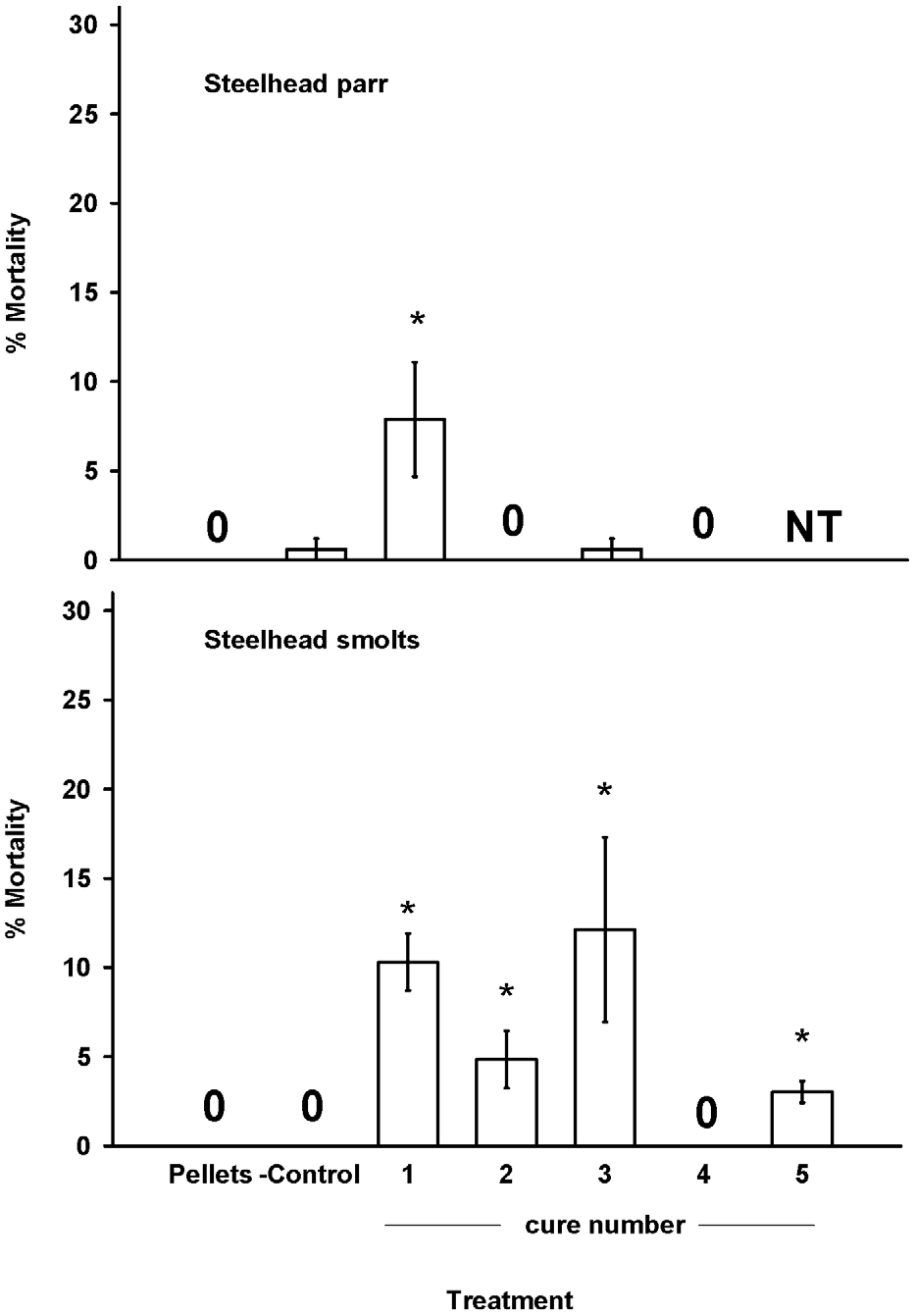
Juvenile steelhead parr and smolt mortality. Juvenile steelhead parr or smolts were fed for 23 d with pellets, salmon eggs (- control), or salmon eggs cured with 1 of 4 or 5 commercially available cures. Bars represent the mean % mortality for three replicate tanks (N = 55 fish per tank). A value of 0 represents treatments that had no mortality in all three tanks. NT = Not Tested *Significantly different from the group fed pellets (*P*<0.05).

**Figure 2:**
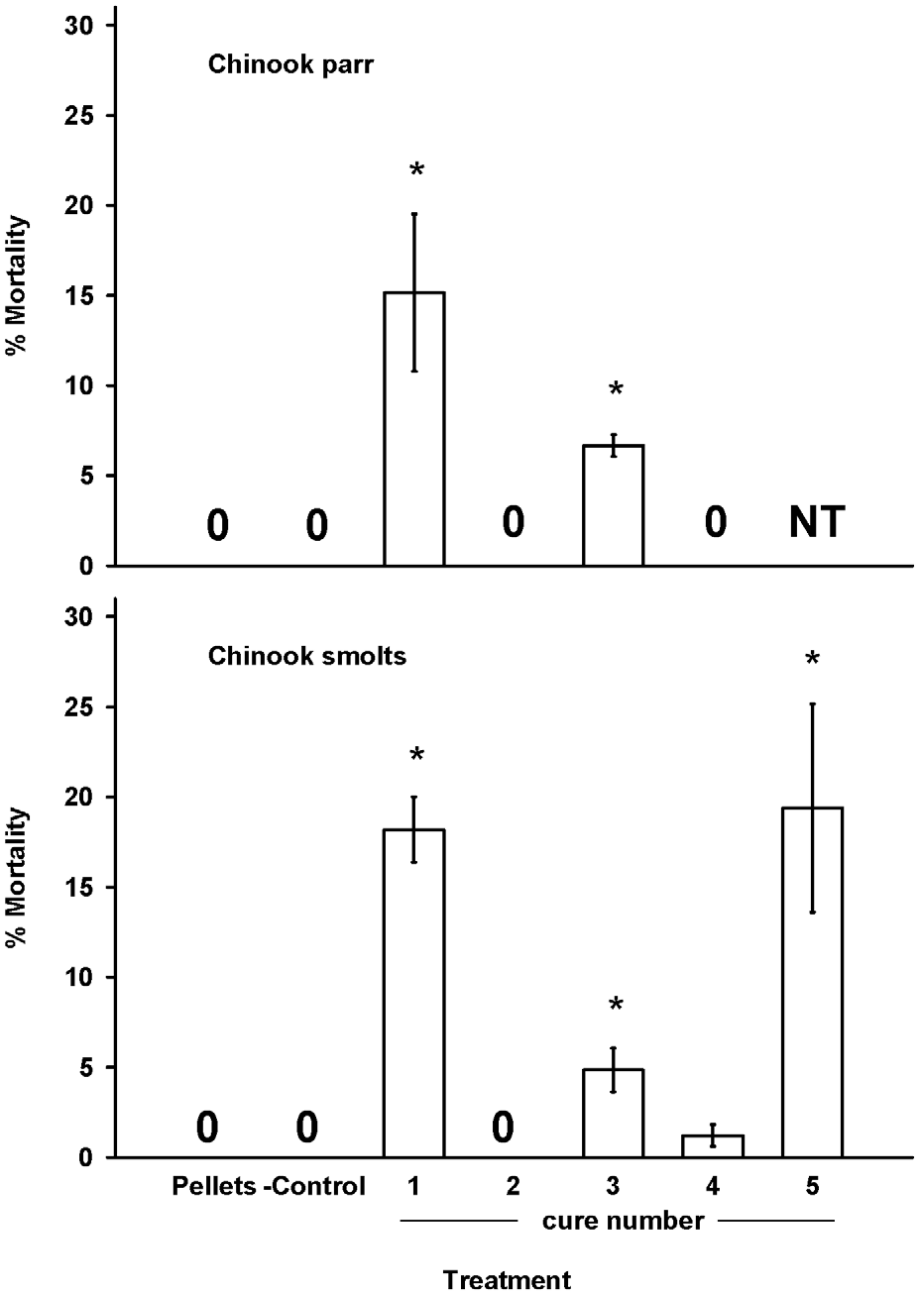
Juvenile spring Chinook parr and smolt mortality. Juvenile juvenile spring Chinook parr or smolts were fed for 23 d with pellets, salmon eggs (- control), or salmon eggs cured with 1 of 4 or 5 commercially available cures. Bars represent the mean % mortality for three replicate tanks (N = 55 fish per tank). A value of 0 represents treatments that had no mortality in all three tanks. NT = Not Tested. *Significantly different from the group fed pellets (*P*<0.05).

In general, the fish fed well during the experiments, though there was some variation in feeding behavior among the treatment groups. We observed very little mortality in both the control and negative control groups. Only one fish from these two groups died during the experiments (from a total of 1320 individuals). This fish had no eggs in its stomach and we were unable to determine the cause of death. In all four trials we observed significant differences in mortality among the groups (P<0.001, ANOVA). Consumption of cure 1 was associated with a significant increase in mortality (P<0.05, Tukey's pairwise comparison) in steelhead parr and smolts (Fig. 1). Similarly, consumption of cures 2, 3, and 5 was associated with a significant increased in mortality (P<0.05, Tukey's pairwise comparison) in steelhead smolts, but not parr (Fig. 1). Spring Chinook parr that consumed cures 1 and 3 had significantly greater mortality than the groups fed pellets (P<0.05, Tukey's Pairwise comparison) (Fig. 2). Similarly, consumption of cures 1, 3, and 5 was associated with a significant increase in mortality (P<0.05, Tukey's pairwise comparison) in the spring Chinook smolts (Fig. 2).

**Figure 3:**
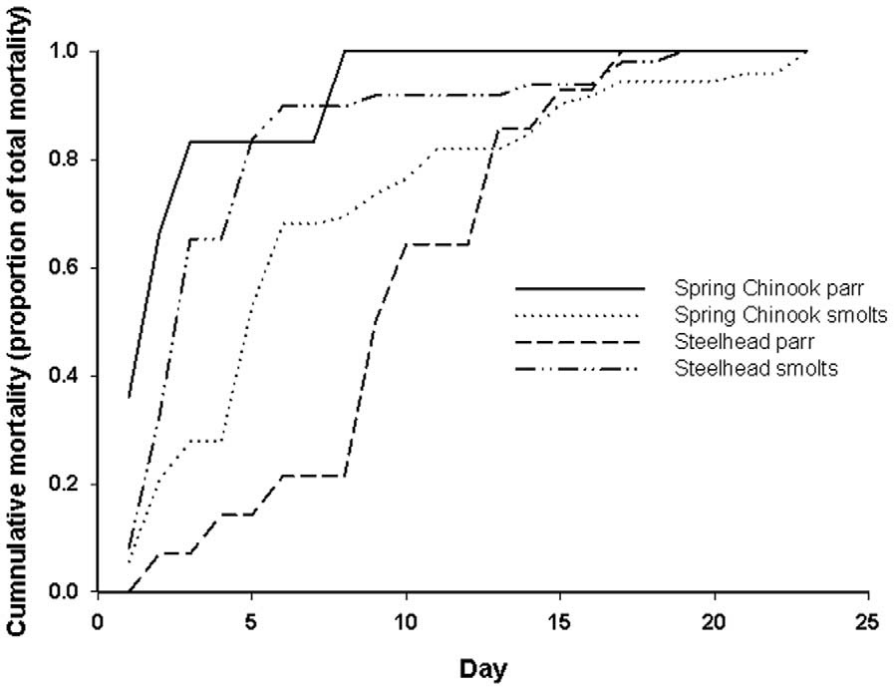
Index of cumulative mortality in juvenile Chinook and steelhead parr and smolts. Juvenile Chinook and steelhead parr and smolts were fed cured eggs for a period of 23 d. We calculated cumulative mortality by summing the number of fish that died each day for all treatment groups (calculated separately for each lifestage and species) fed cured eggs and dividing this by the total number of fish that died during the 23 d period.

In general, the majority of fish died within the first 10 d of feeding. In several instances, fish died after 1 or 2 feedings (Fig. 3). The number of eggs in the stomach of dead fish ranged from 1 to 46 (Table 2). Smolts of both species tended to have more eggs in their stomachs at the time of death than parr. We were unable to confirm whether all fish consumed eggs on any given day. We noted significant tissue degradation in the muscle of the body wall surrounding the gut cavity and of the internal organs in several fish. We collected samples of liver, stomach, intestine, heart, and spleen tissue from surviving fish for histology but found no evidence of tissue damage in these fish.

**Table 2:** Post-mortem evaluation of cured egg consumption.

	steelhead		Chinook	
	parr	smolt	parr	smolt
control	N/A	N/A	N/A	N/A
uncured eggs	0.00	N/A	N/A	N/A
cure 1	4.85	15.06	3.96	5.23
cure 2	N/A	14.00	N/A	N/A
cure 3	10.00	22.15	4.45	3.88
cure 4	N/A	N/A	N/A	1.00
cure 5	N/A	8.60	N/A	2.34

Values represent the mean number of eggs in the gut of fish that died following consumption of eggs treated with 1 of 4 or 5 commercially available cures. N/A indicates no mortality for that lifestage and treatment group.

### 3.2 Effect of sodium sulfite on the survival of juvenile spring Chinook

**Figure 4:**
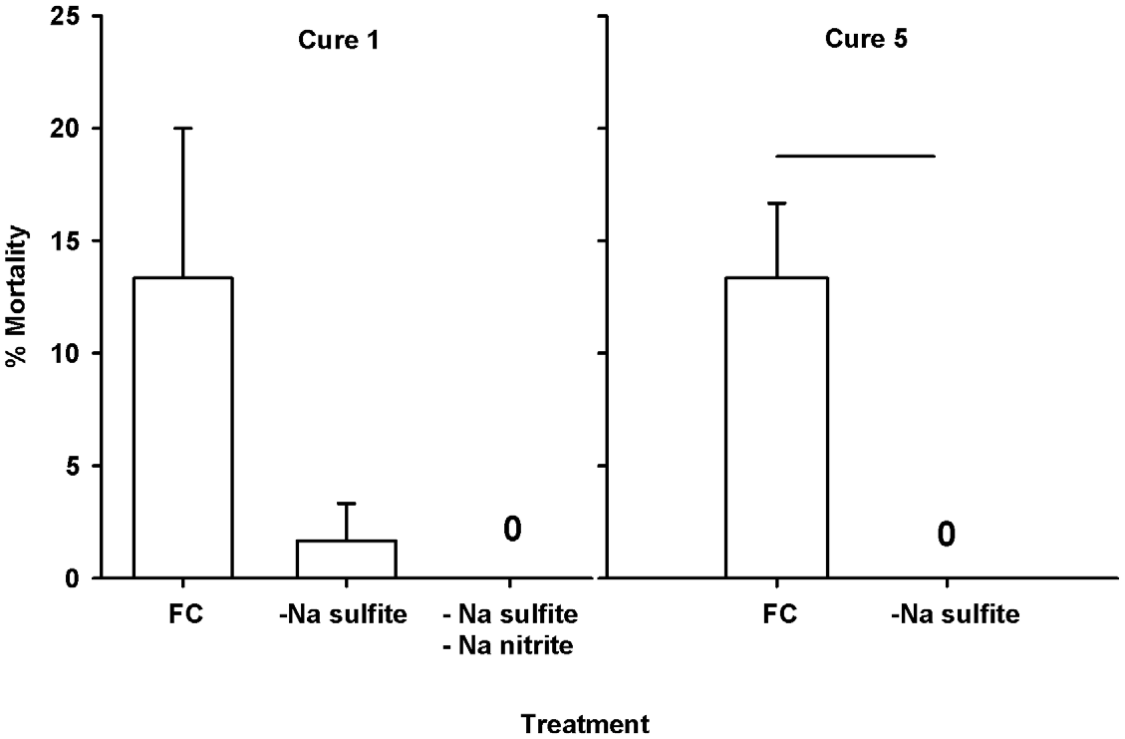
Effect of sodium sulfite on mortality in juvenile spring Chinook. The fish were fed (10 d, 1.5% BW/d) eggs that were cured with the full cure (FC), the same cure without sodium sulfite (-Na sulfite), or the same cure without sodium sulfite or sodium nitrite (-Na sulfite -Na nitrite). We evaluated the effect in two cures (cure 1 and 5), however cure 5 does not contain sodium nitrite. Each bar represents the mean % mortality in 2 (cure 1) or 3 (cure 5) replicate tanks (N = 30 fish/tank). 0 represents no mortality in a tank. Groups sharing a line above the bar are significantly different (*P*<0.05).

The presence of sodium sulfite (or sodium nitrite) did not have a significant effect on mortality in the fish fed cure 1 (P = 0.123, ANOVA). Mean mortality was 11.66%, 1.11%, and 0% in the groups fed the full cure, the cure without sodium sulfite, or the cure without sodium sulfite and sodium nitrite, respectively (Fig. 4). However, the removal of sodium sulfite was associated with a significant (P = 0.001, t-test) decrease in the level of mortality in fish fed cure 5 (mean mortality: 13.33±3.33% and 0% for fish fed cures with and without sodium sulfite, respectively).

The removal of sodium sulfite was also associated with a significant decrease in mortality in both experiments where the cure was directly administered via syringe. For cure 1, mortality was 30% in the group given the full cure and 0% in the group given the cure without Na_2_SO_3_ (P = 0.020, Fisher's Exact Test). Similarly, for those fish fed cure 5, mortality was 35% in the group given the full cure and 0% in the group given the cure without Na_2_SO_3_ (P = 0.008, Fisher's Exact Test).

### 3.3 The relationship between the number of cured eggs consumed, the consumption of alternative food, and the survival time in juvenile spring Chinook

**Figure 5:**
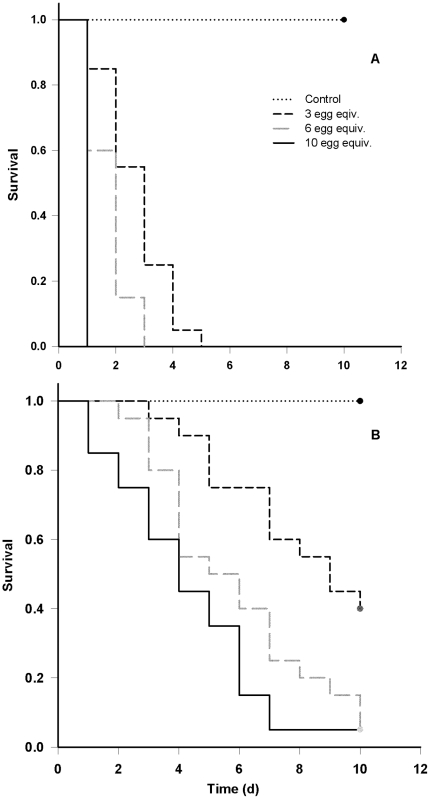
Relationship between the number of eggs consumed and the time to mortality. Survival time of juvenile spring Chinook fed the equivalent of 3 (low), 6 (medium), or 10 (high) eggs that were cured with cure 5 (A) or cure 4 (B). The cured egg mixture was administered into the stomach daily using a syringe. Control fish were given uncured eggs. The experiment was terminated after 10 d. Each group consisted of 20 fish.

The concurrent consumption of pellets had no effect on the survival times in either experiment (Cure 1: P = 0.497; Cure 4: P = 0.286). Similarly, we found no evidence for a tank effect in either experiment (Cure 1: P = 0.889; Cure 4: P = 0.091). The survival time was significantly correlated with the number of eggs consumed in both experiments (Cure 1: P<0.001; Cure 4: P<0.001; Fig. 5). All fish that consumed the equivalent of 10 eggs cured with cure 1 died during the first day. This was significantly faster than the groups consuming the equivalent of 6 eggs (100% mortality within 3 d) and 3 eggs (100% mortality within 5 d). The fish consuming cure 4 survived longer than those consuming cure 1. We observed 100% mortality within 7 d for the fish consuming the equivalent of 10 eggs cured with cure 4. This was significantly faster than the groups consuming the equivalent of 6 eggs (95% mortality within 10 d) and 3 eggs (60% mortality within 10 d). There was no difference in survival time between the HD and MD groups that were administered cure 4.

### 3.4 Effect of intra-buccal administration of the cure

There was no mortality in either treatment group.

### 3.5 Effect of soaking on the toxicity of the cured eggs

**Figure 6:**
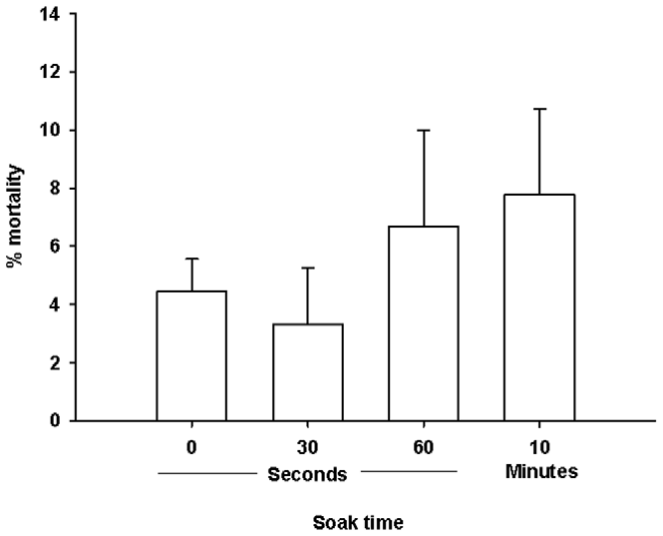
Effect of pre-soaking the cured eggs on the mortality of juvenile Chinook salmon. Eggs cured with cure 1 were soaked for 0 s, 30 s, 60 s, or 10 min prior to feeding. Each bar represents the % mortality in an individual tank (N = 30 fish per tank).

Feeding behavior was similar among all treatment groups and all groups consumed the majority of the eggs on each occasion. Soaking the eggs prior to feeding had no effect on mortality (P = 0.691, ANOVA). Mean mortality (±S.E.) was 4.44±1.11, 3.33±1.92, 6.67±3.33, and 7.78±2.94% in the control, 30 s soak, 1 min soak, and 10 min soak groups, respectively (Fig. 6).

## Discussion

We showed that consumption of eggs cured with some commercially available cures cause mortality in juvenile salmonids. To our knowledge, this is the first such report of this effect. A study conducted in the 1980s evaluated the effect of borax cured eggs and noted a significant decrease in growth and higher levels of cortisol, but no mortality (Bouck et al., unpublished data). The toxic effect in our study appears to be associated with the presence of sodium sulfite in the cures. Rinsing the eggs prior to feeding, as may occur whilst angling, had no effect on mortality. Therefore, we would not expect that eggs (containing sulfites) that are used in rivers and lakes would be any less toxic when used in a typical manner. Interestingly, the overall level of mortality was considerably lower in the experiment evaluating the effect of soaking than in the other experiments. We suspect that this may have been caused by oxidation of the sodium sulfite during storage as the consistency of the cure tended to change over time, becoming darker and moister. Regardless, that we observed any mortality after soaking the eggs for 10 min suggests that it is not possible to eliminate the toxic effect in this way.

Our data suggest there is a positive correlation between the concentration of sodium sulfite in the egg played and mortality. There was a significant difference in the survival times of fish fed the equivalent number of eggs cured with mixtures containing a high (>50% by weight) and low (∼20% by weight) concentration of sodium sulfite. Our intention was to determine whether the number of eggs needed to cause mortality was within the range of what a wild juvenile might consume. Thus, we did not attempt to quantify the actual dose of sodium sulfite in the mixes that were administered. We initially attempted to quantify the number of eggs required to cause mortality by feeding individual fish a known number of eggs. However, the experiment was unsuccessful as Chinook generally do not feed well when held alone in a tank. Interestingly though, we noted a rapid decrease in “appetite” when the individually housed fish were fed cured eggs. This effect was reversed by switching to uncured eggs. This is consistent with the observations of Rankin [10] showing learned aversion to food that is toxic and suggests that the juvenile salmon may learn to avoid such foods following an initial exposure. However, it is not clear whether this would apply in situations where individuals are competing for food as we did not observe a decrease in “appetite” when fish were housed in groups.

Interestingly, we did not observe any mortality in fish that were fed cure 4 for 23 d. We maintained daily records of feeding behavior and this group consistently scored lowest for appetite. In addition, a large number of uneaten eggs were removed from the tanks at the end of the experiment. Taken together, these observations suggest that, when given a choice, few fish consumed the eggs cured with cure 4 during the feeding trials. We suspect this explains the low level of mortality as we did observe high levels of mortality when cure 4 was administered directly into the gut using a syringe.

We conclude that exposure via the gut alone is sufficient to cause death in juvenile salmonids. Exposure via the gills did not cause any mortality over a 10 d period. We made no attempt to determine the cause of death though the available literature suggests a number of possible pathways, including toxicity to the central nervous system [11], [12], disruption of enzyme activity [13], [14], [15], [16], or oxidative damage [17]. Changes in enzyme activity are unlikely to explain the rapid nature of mortality (<6 h) we observed in several animals following ingestion of cured eggs. However, it is possible that such changes may affect long term fitness. In several instances, we observed degradation of the internal organs and musculature of the gut cavity in the fish that died. In addition, we observed external lesions on ∼5 fish. In the latter individuals, the body wall was apparently “dissolved”. This is consistent with observations in other vertebrates that have shown tissue degradation in the gut. We speculate that the degradation was caused by the formation of sulphurous acid. Sulphurous acid is formed by the equilibrium reaction of sulpher dioxide (an intermediary in the breakdown of sodium sulfite) and water. Taken together, these results suggest that the fish may have died from multiple causes, including tissue breakdown, neurotoxicity, inhibition of enzyme activity, or oxidative stress.

Within the body, sulfite is oxidized to the sulfate ion by sulfite oxidase. We hypothesize that the variability in susceptibility among species and lifestages was caused, in part, by differences in the expression of this enzyme. Likewise, up regulation of sulfite oxidase may explain the decrease in mortality after ∼10 d exposure. Interestingly, in some instances (∼30) we observed mortality after a single feeding of the cured eggs. Of these, 4 fish had a single egg in their gut and 13 had between 2–5 eggs in their guts. This suggests that some fish are particularly sensitive to the negative effects of some cures. Furthermore, it is not unrealistic to expect that wild juveniles would consume 1–5 eggs one or more times during their residence in freshwater.

In addition to sodium sulfite, some cures may also contain other sulfites such as sodium metabisulfite or sodium bisulfite. The available toxicity data (http://www.pesticideinfo.org/Search_Chemicals.jsp) suggests that other forms of sulfite may be equally as toxic to fish. For example, the average LC50 for sodium sulfite is 660 mg/l in the western mosquitofish (*Gambusia affinis*) whereas the average LC50 for sodium bisulfite is 240 mg/L (static waterborne exposure). We were not able to find any data for salmonids. Interestingly, sodium nitrite, another ingredient in some cures, appears to be more toxic (LC50: 2.47 mg/L in Chinook salmon) than either of the sulfites. Given the available data, we would urge caution in using any form of sulfite or nitrite without further testing to determine the effects on juvenile fish.

Because the effect appears to be due to the breakdown of chemicals contained within the cured eggs while in the gut, a number of factors may influence the effect in the wild. These include, water temperature and water hardness as well as the presence of other food types in the gut. Temperature is likely to have an effect on both the rate of the chemical reactions and the activity of enzymes, such as sulfite oxidase, which break down sulfites in the body. We found no evidence that the presence of other food in the gut affected the toxicity of the cured eggs. However, we did not test the full range of foods that might be consumed by salmonids, nor did we look at whether prior consumption of alternative foods had any benefit.

In summary, we showed that some commercially available cures killed juvenile salmonids in a laboratory setting. There appears to be a dose dependent effect that is not ameliorated by pre-soaking the eggs prior to feeding. Surprisingly, consumption of relatively few eggs (1–5) was sufficient to cause mortality in some individuals. We have no data to suggest that this does, or does not, represent a problem at the population level. We would further caution that it is not realistic to transfer the rates of mortality observed in this study into the wild. We cannot rule out the possibility that the levels were exacerbated by stress associated with the experimental procedures, particularly with the oral administration studies. Regardless, we believe it is likely that a proportion of juvenile salmonids that consume eggs cured with sulfites will suffer mortality in the wild. Given that the most dominant fish in a population tend to monopolize food resources [18], [19], [20], it is reasonable to speculate that the more dominant fish could be more prone to mortality. Given the risk, we recommend that anglers take steps to minimize this risk. These may include the use of spawn sacs, using cures that do not contain sulfites, and/or avoiding discarding unused baits into the river. In addition, because sodium sulfite was not commonly used in cures prior to 1980 and can likely be replaced by other mold inhibitors, such as borax, we suggest management agencies and manufacturers consider approaches that would minimize incidental take of wild juveniles salmonids when fishing with cured eggs. These might include eliminating the use of sodium sulfite or establishing a level of acceptable risk that is consistent with other forms of indirect fishing related mortality (e.g. hooking mortality).We urge caution when using this approach as we have no data on additive effects or the effects of other cure ingredients. It is likely that other components of some cures are as toxic (e.g., sodium metabisulfite) but currently have little or no effect as they are present at lower levels. Any increase in the concentrations of these compounds as a result of decreasing the concentration of sodium sulfite may result in a similar problem. Similarly, there are no data on the sublethal effects of sulfite exposure in juvenile salmon. A number of studies have shown that exposure to sulfites may have sublethal effects in other vertebrates [1]–[4], [21], [22], including alteration of mineral balance [23] and damage to renal cell membranes [24]. We have no data to suggest that ingestion of sulfites alters the ability of juvenile salmon to osmoregulate or maintain proper ion balance. However, given that smolts may be exposed to sulfites prior to ocean entry this should be given some consideration.
